# Imaging infective endocarditis: Adherence to a diagnostic flowchart and direct comparison of imaging techniques

**DOI:** 10.1007/s12350-018-1383-8

**Published:** 2018-07-31

**Authors:** Anna Gomes, Peter Paul van Geel, Michiel Santing, Niek H. J. Prakken, Mathilde L. Ruis, Sander van Assen, Riemer H. J. A. Slart, Bhanu Sinha, Andor W. J. M. Glaudemans

**Affiliations:** 1 Department of Medical Microbiology, University of Groningen, University Medical Center Groningen, Hanzeplein 1 (HPC:EB80), 9713GZ Groningen, The Netherlands; 2 Department of Cardiology, University of Groningen, University Medical Center Groningen, Groningen, The Netherlands; 3 Department of Radiology, University of Groningen, University Medical Center Groningen, Groningen, The Netherlands; 4 Carl von Ossietzky University Oldenburg, Oldenburg, Germany; 5 Department of Internal Medicine, Infectious Diseases, Treant Care Group, Hoogeveen, The Netherlands; 6 Department of Nuclear Medicine & Molecular Imaging, University of Groningen, University Medical Center Groningen, Groningen, The Netherlands; 7 Department of Biomedical Photonic Imaging, TechMed Centre, University of Twente, Enschede, The Netherlands

**Keywords:** Echo, CT, PET, valvular heart disease, infection, diagnostic and prognostic application

## Abstract

**Background:**

Multimodality imaging is recommended to diagnose infective endocarditis. Value of additional imaging to echocardiography in patients selected by a previously proposed flowchart has not been evaluated.

**Methods:**

An observational single-center study was performed. Adult patients suspected of endocarditis/device infection were prospectively and consecutively enrolled from March 2016 to August 2017. Adherence to a diagnostic imaging-in-endocarditis-flowchart was evaluated in 176 patients. Imaging techniques were compared head-to-head in 46 patients receiving echocardiography (transthoracic plus transesophageal), multi-detector computed tomography angiography (MDCTA), and ^18^F-fluorodeoxyglucose positron emission tomography (FDG-PET/CT).

**Results:**

69% of patients (121/176) adhered to the flowchart. Sensitivity of echocardiography, MDCTA, FDG-PET/CT in patients without prosthesis was 71%, 57%, 29% (86% when combined), while specificity was 100%, 75%, 100%, respectively. Sensitivity in patients with prosthesis was 75%, 75%, 83%, respectively (100% when combined), while specificity was 86% for all three modalities. Echocardiography performed best in the assessment of vegetations, morphological valve abnormalities/dehiscence, septum defects, and fistula formation. MDCTA performed best in the assessment of abscesses and ventricular assist device infection. FDG-PET/CT performed best in the assessment of cardiac device infection, extracardiac infectious foci, and alternative diagnoses.

**Conclusions:**

This study demonstrates that the evaluated imaging-in-endocarditis-flowchart is applicable in daily clinical practice. Echocardiography, MDCTA, and FDG-PET/CT provide relevant complementary diagnostic information, particularly in patients with intracardiac prosthetic material.

**Electronic supplementary material:**

The online version of this article (10.1007/s12350-018-1383-8) contains supplementary material, which is available to authorized users.

## Introduction

Infective endocarditis is a life-threatening disease.^1^ Mortality rates are 15% to 20% during the acute phase and 40% within 1 year.^1^,^2^ Neither the mortality of endocarditis nor its incidence decreased in the past 30 years.^3^ Currently, the incidence of important risk factors is increasing, e.g., aging population, implantation of intracardiac prosthetic material, and healthcare contact.^4^–^9^

Early and accurate diagnosis of endocarditis is crucial, because delay in adequate treatment impairs outcome.^10^,^11^ However, coming to a diagnosis is often difficult and requires a multidisciplinary collaborative approach. Therefore, the clinical diagnosis of endocarditis in everyday practice is based on probability criteria that allow for standardization (European Society of Cardiology [ESC] 2015 modified criteria).^12^

The former (modified Duke) criteria still bear a high degree of diagnostic uncertainty regarded as suboptimal, in particular in patients with intracardiac prosthetic material.^12^–^15^ Therefore, newer imaging techniques, in addition to echocardiography, are now part of the diagnostic workup for endocarditis.^12^ These techniques include computed tomography (CT), ^18^F-fluorodeoxyglucose positron emission tomography/low-dose CT (FDG-PET/CT), and leukocyte scintigraphy with single-photon emission computed tomography/low-dose CT.

**Figure 1:**
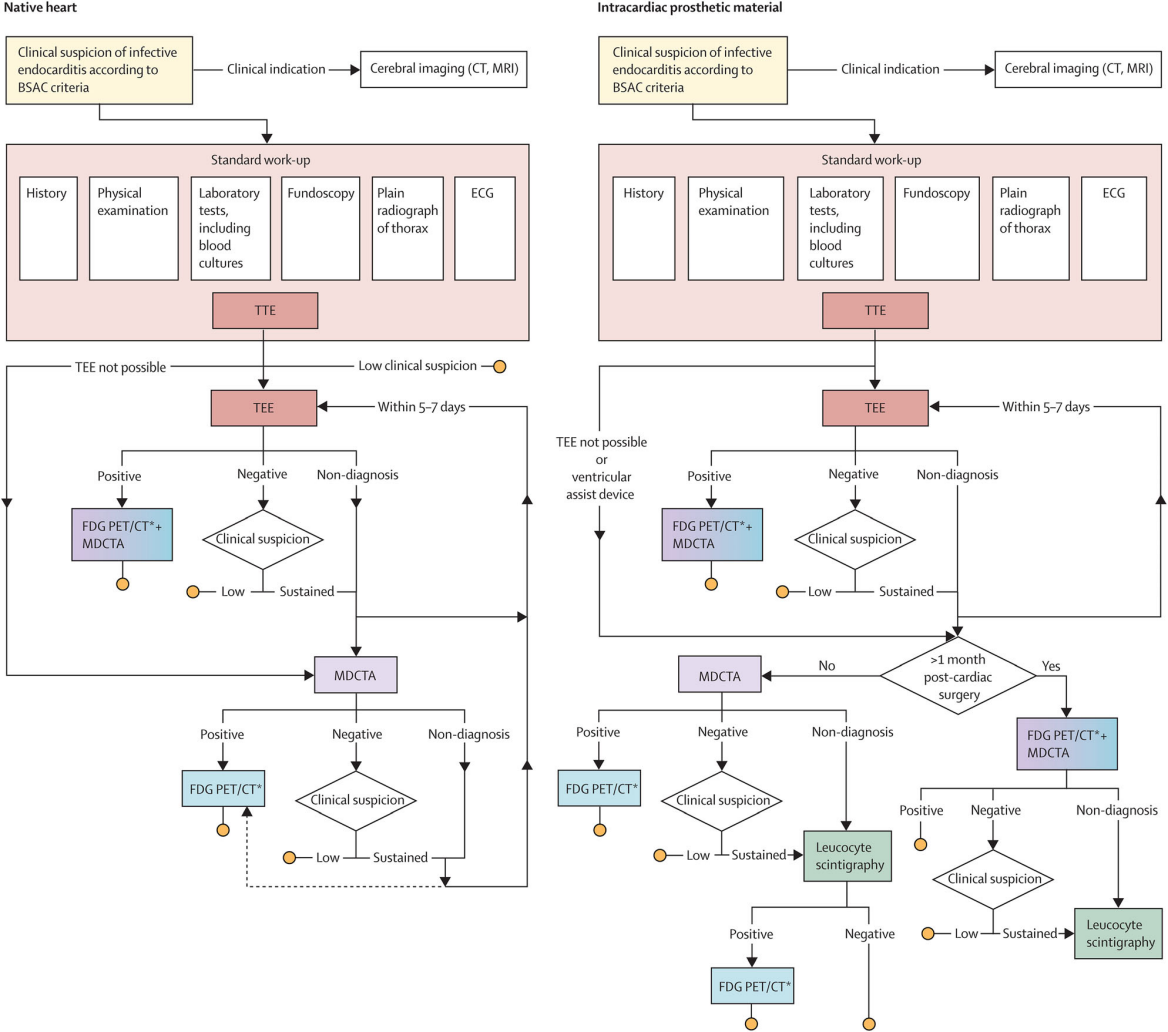
Diagnostic imaging-in-endocarditis-flowchart^16^. Reprinted from The Lancet Infectious Diseases, 17(1), Gomes A, Glaudemans AW, Touw DJ, van Melle JP, Willems TP, Maass AH et al., Diagnostic value of imaging in infective endocarditis: a systematic review, e1–e14, Copyright (2017), with permission from Elsevier

Recently, we published a systematic literature review on the diagnostic value of these newer imaging techniques in endocarditis/device infection, and proposed a diagnostic flowchart (Figure 1)^16^. We hypothesized that this flowchart is applicable in clinical practice. In this study, we evaluated the adherence of all included patients to this flowchart after its implementation in an academic medical center. The available data on prospective head-to-head comparison of imaging techniques in diagnosing endocarditis/device infection is scarce.^16^–^18^ We hypothesized that echocardiography, FDG-PET/CT and electrocardiogram (ECG)-gated multi-detector CT angiography (MDCTA) provide complementary diagnostic information in suspected endocarditis/device infection if their performance is indicated by the flowchart. We compared the accuracy of these techniques head-to-head in a subset of patients refered for all these imaging techniques.

## Material and Methods

### Study Design, Subjects

**Figure 2:**
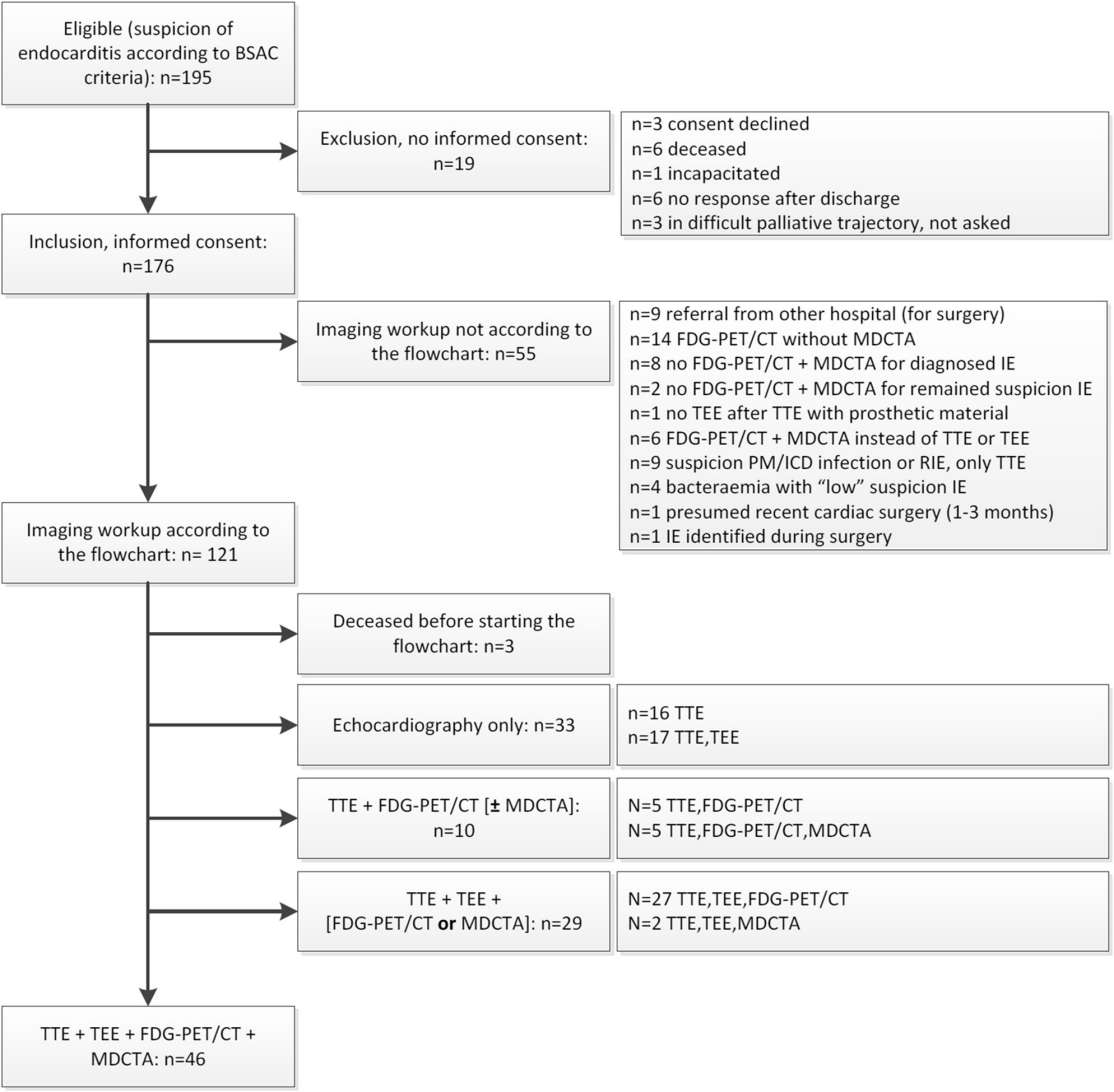
Flow of patients. *BSAC*, British Society for Antimicrobial Chemotherapy; *ICD*, implantable cardioverter defibrillator; *IE*, infective endocarditis; *MDCTA*, multi-detector computed tomography angiography; *PET*, ^18^F-fluorodeoxyglucose positron emission tomography/low-dose CT; *PM*, pacemaker; *RIE*, right-sided endocarditis; *TEE*, transesophageal; *TTE*, transthoracic echocardiography

We performed a prospective observational monocenter study in an academic hospital. The study was approved by the institutional review board (METc2016/045) and subjects signed informed consent. From March 2016 to August 2017 all adult patients presenting with a suspicion of endocarditis/device infection according to “the British Society for Antimicrobial Chemotherapy criteria”^19^ were consecutively enrolled for the flowchart evaluation (Table 1, Figure 2). The inclusion of patients started after the implementation of the flowchart in our hospital protocol for infective endocarditis. Patients receiving FDG-PET/CT, MDCTA, transthoracic (TTE), and transoesophageal (TEE) echocardiography were included in a head-to-head comparison of imaging accuracy for infective endocarditis and/or infection of any component of intracardiac prosthetic material. Patients were treated according to current guidelines^12^,^19^,^20^ and expert opinion.

**Table 1:** Data of included patients for evaluation of the adherence to the imaging-in-endocarditis-flowchart (n = 176)

N = 176	No intracardiac prosthetic material	Intracardiac prosthetic material	Total
Included patients	100	76	176
*n = 105 males (60%), mean age 64 years [18–95], n = 28 deceased (16%)*			
Intracardiac prosthetic material	N/A	76 (100%)	76 (43%)
Valvuloplasty		9 (12%)	9 (5%)
Prosthetic valve (sole)		37 (49%)	37 (21%)
Bentall procedure		8 (11%)	8 (5%)
Pacemaker/ICD		22 (29%)	22 (13%)
LVAD		6 (8%)	6 (3%)
Patch		3 (4%)	3 (2%)
TTE, n (%)	95 (95%)	71 (93%)	166 (94%)
TEE, n (%)	67 (67%)	52 (68%)	119 (68%)
FDG-PET/CT			
Total, n (%)	70 (70%)	49 (64%)	119 (68%)
Cardiac*, n (%)	57 (57%)	45 (59%)	102 (58%)
MDCTA, n (%)	36 (36%)	31 (41%)	67 (38%)
Imaging workup according to flowchart, n (%)	77 (77%)*	44 (59%)*	121 (69%)
*n = 71 males (59%), mean age 65 years [20–95], n = 25 deceased (21%), mean hospital stay 56 days [0–94]*			
Imaging workup not according to flowchart, n (%)	23 (23%)*	32 (41%)*	55 (31%)
*n = 33 males (60%), mean age 61 years [18–84], n = 3 deceased (5%), mean hospital stay 33 days [12–94]*			
Head-to-head comparison	27 (27%)	19 (25%)	46 (26%)
*n = 27 males (59%), mean age 66 years [27–95], n = 5 deceased (11%)*			
Deceased, n (%)	15 (15%)	13 (17%)	28 (16%)

*Deceased*, patient deceased after median follow-up time of 7 months [range 0–15]; *ICD*, implantable cardioverter defibrillator; *LVAD*, left ventricular assist device; *MDCTA*, electrocardiogram-gated multidetector computed tomography angiography; *n*, number of patients; *N*/*A*, not applicable; *FDG-PET/CT total*, ^18^F-fluorodeoxyglucose positron emission tomography with low-dose computed tomography for attenuation correction; FDG-PET/CT cardiac*, good quality PET for cardiac evaluation performed after adequate patient preparation with 24 hour low-carbohydrate, fat-allowed diet and ≥ 6 hour fasting before the scan; *SD*, standard deviation; *TEE*, transesophageal echocardiography; *TTE*, transthoracic echocardiography

*Difference of *P *< 0.05 between the patients with and without intracardiac prosthetic material

### Intracardiac Prosthetic Material

Included prosthesis were valve plasty, biological or mechanical prosthetic valve (sole valve or valve with vascular prosthetic graft), pacemaker, implantable cardioverter defibrillator (ICD), left ventricular assist device (LVAD), and patches to close cardiac defects.

### Reference Standard

The final diagnosis of endocarditis/device infection was established by expert clinical judgement in a multidisciplinary team of endocarditis specialists having access to all available clinical information during hospital admission (with initial interpretation) and follow-up of at least 2 months. The core team consisted of infectious diseases specialists, medical microbiologists, and cardiologists. Guiding in decision making were the ESC 2015 modified criteria,^12^, but ultimately decisive was the complete clinical evaluation and final post-hoc judgement by the expert team.

### Flowchart Adherence

The rating, whether the imaging workup for each patient adhered to the imaging-in-endocarditis-flowchart or not, was determined by expert clinical judgement according to the following rules: (1) the need to perform additional imaging mainly depended on the persistent suspicion of endocarditis/device infection and the existence of a plausible alternative working diagnosis. Following the diagnostic flowchart does not necessarily mean that all of the included imaging techniques needed to be performed; (2) if a technique could not be performed due to contraindications, but the rest of the flowchart was followed, this was regarded as “according to the flowchart”; and (3) if not all planned techniques were performed because a plausible alternative working diagnosis was identified during the diagnostic process, this was regarded as “according to the flowchart”.

### Echocardiography

TTE and TEE were performed according to current guidelines.^14^ Findings that were regarded compatible with a diagnosis of endocarditis/device infection included vegetations, destructive lesions provoking valve aneurysm, perforation, prolapse, chordae or papillary muscle rupture, abscess, pseudoaneurysm, and/or fistula formation. Complications regarded indicative for endocarditis included severe valve regurgitation.

### MDCTA

MDCTA was performed on the same camera system as used for FDG-PET/CT (one-stop-shop principle) after intravenous Iomeron 350 contrast infusion (flow of 4 cc/seconds; volume individually adjusted based on duration of scanning and interscan delay; estimated effective dose of 3 to 10 mSv). Retrospective ECG-gating at 30% to 70% was used. Subsequently, all anonymized scans were analyzed individually, seperately and independently by two readers (MS, NHJP) who were blinded for all clinical information. A locally applied, predefined scoring system for the assessment of CTA was used for findings consistent with endocarditis/device infection. These signs included vegetations, destructive lesions provoking valve aneurysm, perforation, prolapse, chordae or papillary muscle rupture, abscess, pseudoaneurysm, and/or fistula formation. Any inconsistencies were resolved in consensus. The final diagnosis was reported as either “positive” or “negative”, but in some cases both readers indicated that additional information was necessary and these scans were additionally classified as “possible”.

### FDG-PET/CT

FDG-PET/CT was performed with the same camera system as used for MDCTA (one-stop-shop principle). According to the flowchart, FDG-PET/CT could be indicated for the cardiac diagnosis of endocarditis/device infection or to identify extracardiac infectious foci. For all scans, patients were prepared with a 24-hour low-carbohydrate/fat-allowed diet and a minimum of 6 hours fasting. All patients were scanned from skull-base to mid-thigh. Images were acquired on a BioGraph 64-slice mCT (Siemens Healthcare, Knoxville, USA) and reconstructions were performed according to the EANM/EARL guidelines.^21^ FDG-activity of 3 MBq/kg of body weight (mean 244 MBq ± 53 MBq) was injected intravenously 60 minutes before PET data acquisition, according to existing guidelines.^21^ All scans were accompanied by low-dose CT-scanning for attenuation correction and anatomical positioning and performed early in the diagnostic process, preferably within 4 days and maximally 7 days after the start of antimicrobial therapy. All anonymized scans were seperately analyzed in consensus by two experienced readers (AWJMG, RHJAS) who were blinded for all clinical information. Image analysis was performed using the Siemens Syngo.via (Client version 3.0; Siemens, Erlangen, Germany). FDG-uptake was evaluated qualitatively by pattern recognition (homogeneous or focal/heterogeneous) and by a predefined visual grading system using a 4-point score (1:uptake < mediastinum, 2:liver > uptake > mediastinum, 3:uptake = liver, 4:uptake > liver). Liver function (ASAT/ALAT) was checked to decide if liver FDG-uptake could be used as a reference.^22^ Uptake was graded as “positive” for infection when tracer uptake intensity was graded ≥ 2 and when the pattern was focal/heterogeneous.

### Statistical Analysis

The sensitvity of two different imaging workups (echocardiography alone *vs.* with FDG-PET/MDCTA) was compared within groups (with/without prosthesis) with two-sided McNemar’s testing of paired proportions. Differences between groups were compared with the two-tailed Fisher’s exact and unpaired *t* tests. *P* values < 0.05 are suggestive of a difference between groups.

## Results

### Flowchart Adherence

The majority (121, 69%) of 176 enrolled patients received an imaging workup according to the flowchart (Figure 2, Table 1). In patients with prosthetic material, compliance with the flowchart was significantly lower than in patients without prosthetic material (59% vs 77%, *P *= 0.0086), but there were no statistical differences between these groups regarding the number of performed imaging procedures (Table 1).

Major reasons for not adhering to the flowchart are shown in Figure 2. Reasons directly after introduction of the flowchart included unfamiliarity with it and—in the beginning—unavailability of a one-stop-shop protocol for concurrent FDG-PET/MDCTA. Other reasons for non-compliance were referral from another hospital, suspicion of right-sided endocarditis (focus on TTE and not TEE), suspicion of LVAD driveline infection (focus on FDG-PET/CT), reluctance to use FDG-PET/CT within 1 to 3 months after cardiothoracic surgery and performance of FDG-PET/CT for *Staphylococcus aureus* bacteraemia which routinely neither includes appropriate patient preparation nor MDCTA.

Other reasons for refraining from more imaging, but regarded as “in accordance with the flowchart”, included patient death before completion of the workup, indication for emergency surgery, and inability to perform TEE (lack of cooperation, patients’ refusal to perfom the procedure, anatomical abnormalities, or swallowing disorders). Reasons for refraining from MDCTA included contraindication for the use of contrast agents [anaphylactic reaction or renal failure (estimated glomerular filtration rate < 45 mL/min)] and cardiac tachycardia/arrhythmias.

### Head-to-Head Comparison

Twenty-six percent (46/176) of the included patients underwent echocardiography (TTE and TEE), FDG-PET/CT, and MDCTA as indicated by the flowchart (Figure 2, Table 1). In these patients, available for a head-to-head comparison, endocarditis/device infection were more often diagnosed in patients with vs without prosthesis (63% [12/19] vs 26% [7/27], *P *= 0.02, Table 2). Leukocyte scintigraphy was not performed in any patient.

**Table 2:** Data of patients included for a head-to-head analysis of imaging techniques (n = 46)

N = 46	No intracardiac prosthetic material	Intracardiac prosthetic material
Patients	27	19
Intracardiac prosthetic material	N/A	19 (100%)
Valvuloplasty	N/A	3 (16%)
Prosthetic valve	N/A	12 (63%)
Biological		8 (42%)
Mechanical		2 (11%)
Bio-Bentall		1 (5%)
Mechano-Bentall		1 (5%)
Pacemaker/ICD	N/A	3 (16%)
LVAD	N/A	2 (11%)
Patch	N/A	2 (11%)
Time since cardiothoracic surgery, median [range]	N/A	2.9 years [9 days–8.4 years]
TTE/TEE positive, n (%)	5 (19%)*	10 (53%)*
MDCTA positive, n (%)	9 (33%)	10 (53%)
FDG-PET/CT positive, n (%)		
Cardiac	2 (7%)*	11 (58%)*
Extracardiac	21 (78%)	13 (68%)
Final diagnosis endocarditis/device infection, n (%)	7 (26%)*	12 (63%)*

*Final diagnosis*, patient diagnosed during expert team meeting after a median follow-up time of 6 months [range 2–17]; *ICD*, implantable cardioverter defibrillator; *LVAD*, left ventricular assist device; *MDCTA*, electrocardiogram-gated multidetector computed tomography angiography; *n*, number of patients; *N*/*A*, not applicable; *FDG-PET*/*CTextracardiac*,^18^F-fluorodeoxyglucose positron emission tomography with low-dose computed tomography for attenuation correction; *FDG-PET*/*CT cardiac*, good quality PET for cardiac evaluation performed after adequate patient preparation with 24 hour low-carbohydrate, fat-allowed diet and ≥ 6 hour fasting before the scan; *TEE*, transesophageal echocardiography; *TTE*, transthoracic echocardiography. *Difference of *P *< 0.05 between the patients with and without intracardiac prosthetic material

#### Diagnostic accuracy

Echocardiography performed better in patients without vs with prosthesis (Table 3). In patients without prosthesis, echocardiography had a better sensitivity and specificity than MDCTA (71% and 100% vs. 57% and 75%, respectively, Table 3). Conversely, in patients with prosthesis, echocardiography and MDCTA had equal sensitivity (75%) and specificity (86%, Table 3). MDCTA yielded more false-positive results in patients without prosthesis and performed better in patients with prosthesis (positive predictive value 44% and 90%, respectively; Table 3). FDG-PET/CT was more sensitive in patients with vs without prosthesis (83% *vs.* 29%, Table 3).

**Table 3:** Diagnostic accuracy of imaging techniques for the cardiac diagnosis of endocarditis/device infection (n = 46)

No intracardiac prosthetic material				
	TTE/TEE	Endocarditis/device infection		
		Yes	No	Total
Sensitivity 71% Specificity 100% PPV 100% NPV 91%	Positive	5	0	5
	Negative	2	20	22
	Total	7	20	27

	MDCTA	Endocarditis/device infection		
		Yes	No	Total
Sensitivity 57% Specificity 75% PPV 44% NPV 83%	Positive	4	5	9
	Negative	3	15	18
	Total	7	20	27

	FDG-PET/CT	Endocarditis/device infection		
		Yes	No	Total
Sensitivity 29% Specificity 100% PPV 100% NPV 80%	Positive	2	0	2
	Negative	5	20	25
	Total	7	20	27

Intracardiac prosthetic material				
	TTE/TEE	Endocarditis/device infection		
		Yes	No	Total
Sensitivity 75% Specificity 86% PPV 90% NPV 67%	Positive	9	1	10
	Negative	3	6	9
	Total	12	7	19

	MDCTA	Endocarditis/device infection		
		Yes	No	Total
Sensitivity 75% Specificity 86% PPV 90% NPV 67%	Positive	9	1	10
	Negative	3	6	9
	Total	12	7	19

	FDG-PET/CT	Endocarditis/device infection		
		Yes	No	Total
Sensitivity 83% Specificity 86% PPV 91% NPV 75%	Positive	10	1	11
	Negative	2	6	8
	Total	12	7	19

**Figure 3:**
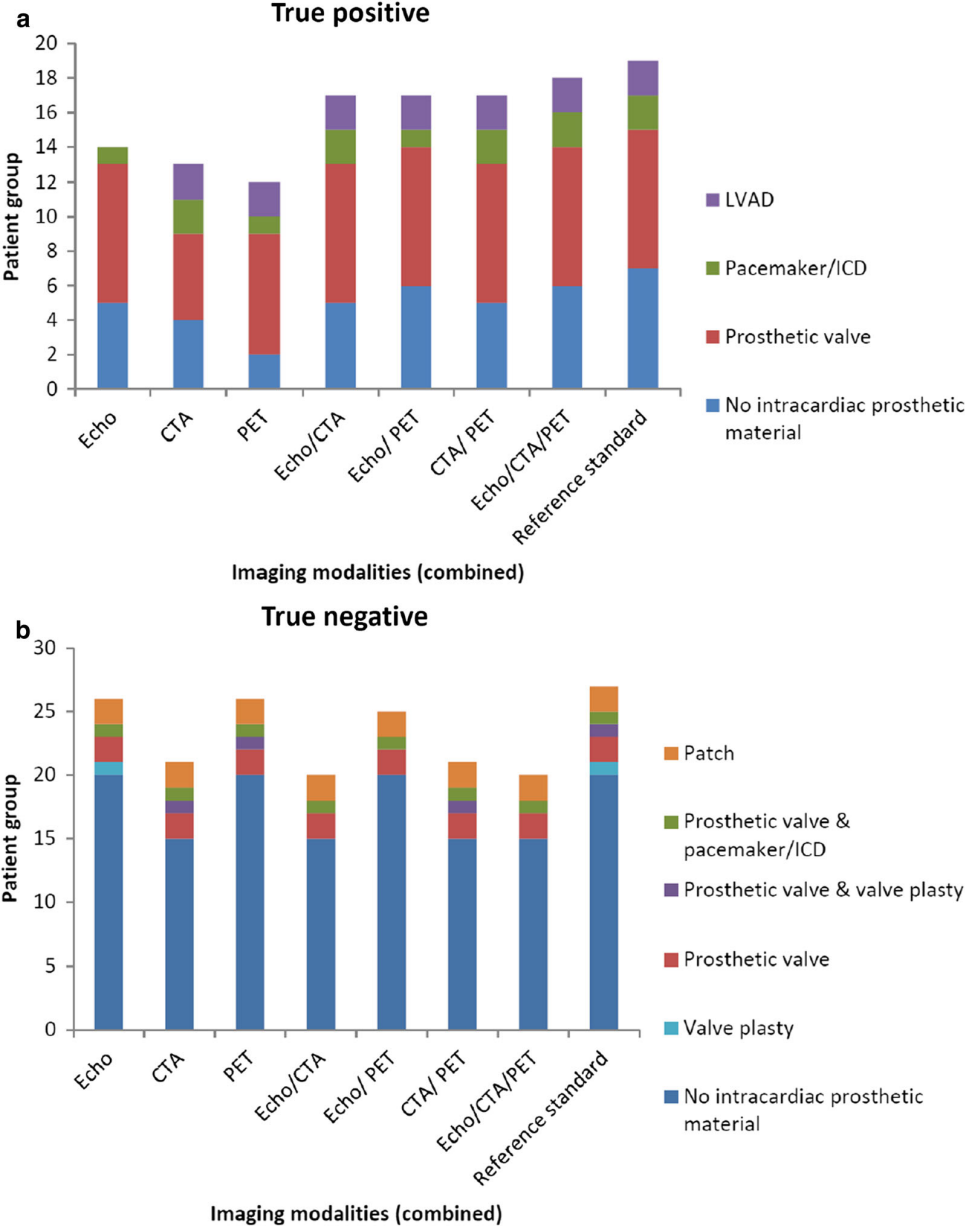
True positive (**A**)/negative (**B**) imaging. Figure shows that maximum sensitivity is reached with all techniques combined, but at the cost of decreased specificity. *CTA*, MDCTA; *Echo*, (transthoracic and transesophageal) echocardiography; *PET*, FDG-PET/CT

Of the 19 patients included in the head-to-head comparison with a final diagnosis of endocarditis/device infection, 73% (14/19) were identified by echocardiography, 68% by MDCTA (13/19), 63% by FDG-PET/CT (12/19), and 95% by all techniques together (18/19) (Figure 3, Supplementary Table S1). The combined use of all techniques identified endocarditis/device infection in 86% of patients without prosthesis (6/7) and 100% of patients with prosthesis (12/12).

#### Relative contribution

**Figure 4:**
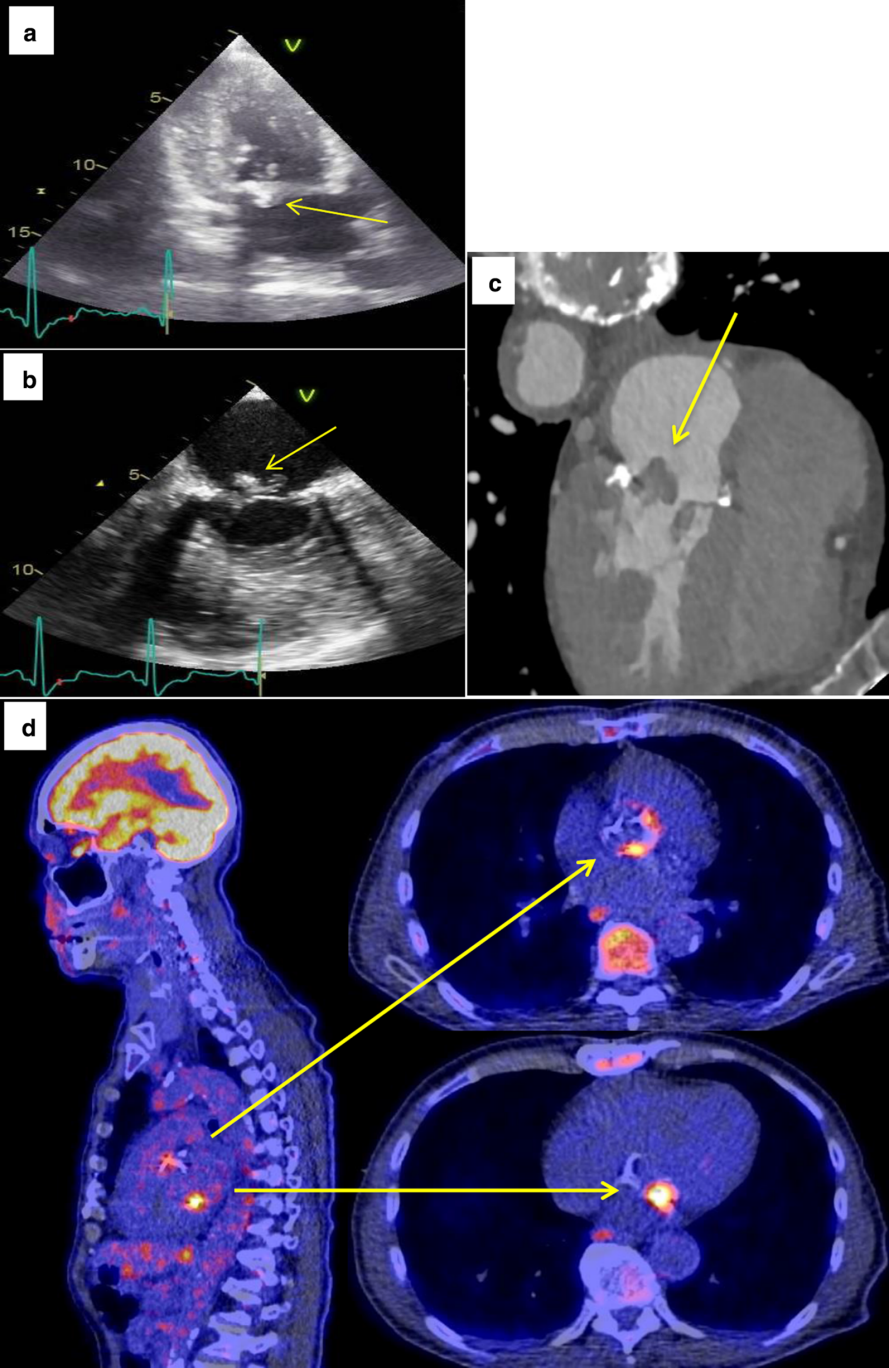
Illustration of the complementary information provided by different imaging techniques. Data shown for a 73-year-old male with *Enterococcus faecalis* endocarditis of his biological prosthetic aortic valve and native mitral valve (Table 4 nr. 23, study nr. 10000246): **A** transthoracic echocardiography, two chamber view, showing the mitral valve with vegetation; **B** transesophageal echocardiography, mitral commissural 60° view, showing the mitral valve with vegetation; **C** contrast-enhanced ECG-triggered MDCTA-scan, four chamber view, showing the mitral valve with vegetation; **D** fused FDG-PET/CT-scan, sagittal and horizontal views, showing FDG-uptake equivocal at the aortic valve (circular) and increased focal at the mitral valve (spot)

Besides the relevance of negative scans in the clinical reasoning process, the addition of both FDG-PET/CT and MDCTA to echocardiography provided clinically relevant information regarding (extent of) the infection in 2 of 7 patients without prosthesis (*P *= 0.480) and in 8 of 12 patients with prosthesis (*P *= 0.013) (Table 4, Figure 4, Supplementary Table S2). Including confirmation of diagnosis, these numbers were 3 of 7 (*P *= 0.248) and 11 of 12 (*P *= 0.003) patients, respectively.

**Table 4:** Discrepancy analysis showing the yield of imaging techniques (n = 29)

N = 29 Nr.	IPM	Pathogen detected	Imaging techniques			Reference standard (incl. surgery)
			TTE/TEE	MDCTA	FDG-PET/CT (visual valve)	
1	Bio-PV	*S. aureus*	TP: MV, PV vegetations with severe PI, small ASD	FN	TP: PV grade 2 focal uptake	P
2	None	*S. aureus*	TN	FP: MV thickening	TN	N
3	None	*T. whipplei*	TP: AV, MV, PV vegetations with severe AS, AI, PI	TP: AV vegetation,abscess; AV, MV, PV thickening	TP: AV grade 4 focal uptake	P (destructed AV, MV)
4	None	*S. aureus*	TN	TN (possible): AV, MV thickening; AV surplus	TN	N
5	Pacemaker	*S. aureus*	FN	TP: MV, AV thickening	FN	P
6	Bio-AV, MVP	*S. aureus*	FP: dehiscence MVP	TN	TN	N (AV normal, MV destructed)
7	None	*S. aureus*	FN	FN	FN	P
8	None	*S. aureus*	TN	FP (possible): AV annular thickening; MV thickening, surplus.	TN	N
9	None	*S. equi*	TN	TN (possible): AV thickening, surplus	TN	N
10	LVAD, MVP	*S. aureus*	FN	TP: air bubbles, contrast extravasation, induration outflow graft, retrosternal abscess/hematoma	TP: LVAD and driveline grade 4 focal uptake	P (indurated tissue around driveline, large cavity around LVAD with 150ml retrosternal pus, fat necrosis around outflow cannula)
11	None	None	TN	TN (possible): AV thickening, surplus.	TN	N (AV calcified)
12	Mechano-Bentall	*P. acnes*	TP: AV paravalvular cavity communicating with LVOT, paravalvular regurgitation	TP: AV vegetation, dehiscence, dysfunction, large abscess, paravalvular leakage. MV thickening	TP: AV grade 4 focal uptake, 2 spleen abscesses	P (dehiscence proximal suture line with large abscess cavity, AV 33% lose)
13	Bio-AV	*S. aureus*	TP: MV vegetations.	FN	TP: AV grade 4 focal uptake, abscess left groin.	P
14	Mechano-MV	*S. epidermidis*	TP: MV annulus vegetations with severe regurgitation	FN-suboptimal scan-	TP: MV grade 3 focal uptake	P
15	None	*S. aureus*	TN	FP (possible): TV thickening, surplus	TN	N
16	None	*S. aureus*	TN	FP: MV thickening, surplus.	TN	N
17	Bio Bentall	*L. monocytogenes*	TP: AV vegetation, annulus thickening, paravalvular regurgitation	TP: AV surplus, annular thickening, fat infiltration. MV thickening, surplus	TP: AV grade 4 focal uptake	P
18	None	*S. mitis*	TP: AV vegetation, thickening; severe AI; pericardial fluid	TP: AV thickening, surplus	FN	P (AV destructed)
19	None	*T. whipplei*	FN	FN (possible): MV thickening, surplus.	TP: MV grade 3 focal uptake	P
20	None	*S. aureus*	TN	FP: MV thickening, surplus	TN	N
21	MVP	None	TN	FP: MV annular thickening, surplus.	FP: MV grade 4 focal uptake	N
22	Bio-AV	*S. aureus*	TP: AV paravalvular abscess	TP (possible): AV surplus, annular infiltration	TP: AV grade 4 focal uptake	P
23	Bio-AV	*E. faecalis*	TP: MV vegetation, prolapse; MS	TP: MV annulus vegetation, degeneration, detachment papillary muscle/chordae; AV annulus thickening	TP: MV grade 4 focal uptake, AV equivocal	P (signs of endocarditis on AV, MV; AV destructed, MV calcified)
24	LVAD	*S. lugdunesis*	FN	TP: LVAD infection	TP: LVAD grade 4 focal uptake, bone metastatic infection of right hip and shoulder, left wrist	P (small hole in ouflow graft with pus in bend relief, surrounding indurated tissue)
25	None	*S. aureus*	TP: AV vegetation, paravalvular abscess, fistula, destruction	TP: AV vegetation and thickening, MV vegetation.	FN	P (AV insufficient, paravalvular abscess)
26	Bio-AV	*P. acnes*	TP: AV vegetations, paravalvular abscess; pericardial fluid	TP: AV thickening, dehiscence, angulation, dilatation	FN	P (prosthesis dysfunction)
27	Pacemaker	*S. aureus*	TP: lead vegetation, slight TI	TP: lead surplus	TP: lead grade 2 focal uptake, metastatic foci in both lungs, bone (spondylodiscitis L2, right hip and shoulder), aortic root, RCA	P
28	None	*S. dysgalactiae*	TP: MV vegetation, AV destruction, severe AI, AS, poor LV function	FN	FN	P (AV calcified)
29	None	*S. mutans*	TP: AV vegetations, perforation, thickening	TP: AV thickening, surplus	FN	P (AV destructed, insufficient, stenotic)

The remaining n = 17 patients had all imaging negative and no endocarditis/device infection

*Gold standard*, expert team diagnosis; *surplus*, non-conclusive vegetation/pannus/thrombus detected; *AI*, aortic valve insufficiency; *AS*, aortic valve stenosis; *ASD*, atrial septum defect; *AV*, aortic valve; *FN*, false negative; *FP*, false positive; *IPM*, intracardiac prosthetic material *in situ*; *LV*, left ventricle; *LVOT*, left ventricular outflow tracts; *MI*, mitral valve insufficiency; *MS*, mitral valve stenosis; *MV*, mitral valve; *MVP*, mitral valve plasty; *N*, negative; *Nr.*, patient number; *P*, positive; *PI*, pulmonary valve insufficiency; *PS*, pulmonary valve stenosis; *PV*, pulmonary valve; *TI*, tricuspid valve insufficiency; *TN*, true negative; *TP*, true positive; *TV*, tricuspid valve

Echocardiography identified valve insufficiency and stenosis, septum defects, pericardial fluid and assessed ventricular function. Valve perforation and destruction (n = 3) and fistula formation (n = 2) were only identified by echocardiography. Echocardiography identified vegetations in 12 of 13 patients and visualized 4 of 5 abscesses. Prosthetic valve dehiscence and regurgitation were identified with echocardiography (2 of 3) as was valve prolapse (1 of 1). Valve plasty dehiscence (1 of 1) however was falsely attributed to endocarditis. Echocardiography was false negative in 5 patients: 2 with LVAD related infection, 2 with native valve endocarditis (NVE), and 1 with an unclear locus of infection (Table 4—nr. 5, study nr. 10000092, who had a cerebral vascular accident).

MDCTA identified vegetations in 8 patients. Notably, MDCTA often identified not further specified valve “surplus” (non-conclusive vegetation/pannus/thrombus), which could retrospectively be identified as a vegetation with a final diagnosis of endocarditis in some cases but as false-positive in others (n = 6). MDCTA identified abscesses (5 of 5), LVAD related infection (2 of 2), prosthetic valve dehiscence and regurgitation (2 of 3) and valve prolapse (1 of 1). Notably, it identified valve thickening neither identified by echocardiography nor FDG-PET/CT, retrospectively to be regarded as a sign of endocarditis in 3 patients but as false-positive in others (n = 7). MDCTA was false negative in 5 patients (including 1 suboptimal scan): 4 had vegetations (3 prosthetic and 1 native valves) and 1 had *T. whipplei* NVE which was only FDG-PET/CT positive. With MDCTA coronary stenosis was identified in 20 of 46 patients (43%).

Physiological myocardial FDG-uptake was sufficiently suppressed in 91% (42/46) of patients. Liver function tests were normal in 93% (43/46) of patients. In the other 3, liver function was affected, but FDG-uptake was regarded within the normal range, thereby not influencing assessment of potential pathological foci. FDG-PET/CT identified LVAD related infection (2 of 2), pacemaker lead infection (1 of 1) and infected valves (3 native, 6 prosthetic valves). FDG-PET/CT was false negative in 7 patients: 5 with NVE, 1 with prosthetic valve endocarditis (PVE) and 1 with an unclear focus of infection (previously described). FDG-PET/CT also identified important septic emboli and metastatic infection in 6 patients, (abscesses in the spleen and groin, metastatic infection of the hip, shoulder, wrist, spine, aortic root, coronary artery and lungs), other alternative foci for infection in 16 patients and other complications (e.g., detection of possible occult primary malignant tumors) in 17 patients.

## Discussion

We evaluated the adherence to an imaging-in-endocarditis-flowchart and showed that it is applicable in clinical practice. We found a difference in the adherence between patients with and without prosthesis, but not in the applied imaging techniques in these groups. Non-adherence was probably due to a similar workup as in patients without prosthesis; while according to the flowchart in patients with prosthesis additional imaging techniques were required. We revealed an optimal sensitivity for patients without prosthesis of 86% and with prosthesis of 100%, when echocardiography, MDCTA and FDG-PET/CT were combined for the diagnosis of endocarditis/device infection and demonstrated that these imaging techniques provide complementary diagnostic information if they are indicated by the flowchart. Adding FDG-PET/MDCTA to echocardiography provides significant relevant information in patients with prosthesis. Therefore, our results support the use of additional imaging techniques as indicated by the flowchart, aiding diagnosis particularly in patients with prosthesis.

In this study, echocardiography performed best in identifying morphological valve abnormalities, septum defects, and fistula formation. It was the only technique assessing ventricular function. Echocardiography was superior to MDCTA for the identification of vegetations and prosthetic valve dehiscence.

Our results confirm that MDCTA is superior to echocardiography for the identification of abscesses and is regarded particularly useful in patients with prosthesis due to their high incidence of abscesses and mycotic aneurysms.^23^ In addition, MDCTA identified all LVAD infections and visualized the coronary arteries. Hereby, it has the potential to improve prognosis by guiding surgical management.^24^ MDCTA generally performed less well in our study as compared to earlier studies reporting a pooled sensitivity, specificity, positive and negative predictive value of 93% to 100%, 83% to 88%, 97%, and 88%, respectively.^24^–^26^ The lower performance in our study likely reflects factors compatible with clinical practice: (1) instead of patients with possible/definite endocarditis according to the modified Duke criteria, we included patients suspected of endocarditis/device infection based on “the British Society for Antimicrobial Chemotherapy criteria”;^19^ (2) instead of 64/256-slice and dual source scanners, we used a 64-slice scanner; and (3) a different assessment of 37% (17/46) scans between more and less experienced readers.

Our data support the combined use of FDG-PET/MDCTA, that can be performed by hybrid camera systems during a single visit. MDCTA was positive while FDG-PET/CT was negative in 5 cases and MDCTA was negative while FDG-PET/CT was positive in 4 cases. We noticed that MDCTA identified not further specified “surplus” on and thickening of valves, falsely regarded compatible with endocarditis/device infection. The functional data provided by FDG-PET/CT distinguishes active from non-active deviations identified by MDCTA, mainly in patients with prosthesis.^27^ In the ESC 2015 modified criteria, abnormal perivalvular FDG-uptake is a major criterion for PVE but not for NVE, due to its low sensitivity in this group.^12^,^16^–^18^,^28^,^29^ Furthermore, FDG-PET/CT can detect extracardiac infectious complications which might reveal an additional minor diagnostic criterion.^12^ In our study, FDG-PET/CT correctly identified PVE in 86% (6/7) of cases, and possibly missed one due to its low-virulent pathogen *Propionibacterium acnes*. In line with literature, FDG-PET/CT indicated for the identification of extracardiac infectious complications or alternative diagnosis, identified NVE in 38% (3/8) of cases.^16^ FDG-PET/CT identified LVAD and pacemaker infection. Three patients with pacemakers were included in the head-to-head comparison and FDG-PET/CT identified extracardiac foci in all. In one patient it additionally showed pathological uptake at the lead, the aortic root and right coronary artery. FDG-PET/CT also demonstrated major clinical importance by imaging the rest of the body, detecting multiple septic emboli, metastatic infection, possible occult primary malignant tumors, alternative infectious foci, and other complications.

In our diagnostic imaging-in-endocarditis flowchart, we advise not to perform FDG-PET/CT during a period of 1 month after surgery. The ESC guidelines of 2017 state a 3-month period post cardiothoracic surgery in which it is advised not to perform FDG-PET/CT for diagnosing infective endocarditis, due to a risk of false-positive results of the regenerative process and post-surgical inflammation. However, the 3 month restriction period as stated in the ESC guidelines is not based on strong scientific evidence. There is still debate ongoing regarding the minimal interval. Both 3 months (by the ESC) and 1 months (by the EANM guidelines for FDG-PET imaging in infectious diseases)^21^ have been proposed. Instead of defining a strict post-operative period before performing FDG-PET/CT, one should always keep in mind the possibility of false-positive findings post cardiothoracic surgery, also depending on used material and surgical glue. This is also the case even years after the implantation. Besides, this post-operative period only accounts for the surgical area; disseminated areas of infection outside the heart region should not have this limitation.

A potential limitation includes selection bias for the head-to-head comparison, as it was performed in more complicated cases in which all imaging was obtained. Nonetheless, as we aimed to evaluate the flowchart, the accuracy of imaging in the patients for whom the flowchart indicates it, is relevant. Patients with a lower suspicion of endocarditis/device infection received clinical care probably to a lower degree guided by the imaging-in-endocarditis-flowchart. As a consequence, selection bias might also explain the difference found in the mortality rates of patients following the flowchart vs patients that did not. Also the limited number of patients in the head-to-head comparison is a limiation, especially the relative large part (27 patients) without intracardiac prosthetic material in which FDG-PET/CT normally is limited and has to be interpreted carefully. However, in this group in a large amount of patients (78%) extracardiac findings were detected on FDG-PET/CT, emphasing the role of this imaging technique also in this patient group. Another limitation includes the reassessment of FDG-PET and MDCTA by observers blinded to the clinical data, which resulted in more conservative estimates than in clinical practice due to their lack of information, thereby reducing external validity.

In summary, this is the first study to investigate the feasability, adherence, and performance of an imaging-in-endocarditis-flowchart in patients suspected of endocarditis/device infection. We conclude that the flowchart is applicable in clinical practice and of added value, as multimodality imaging suggested by the flowchart provides complementary diagnostic information in patients, especially in those with intracardiac prosthetic material. Future studies should assess whether the flowchart conveys a better prognosis for patients and cost-effectiveness of this diagnostic algorithm.

## New Knowledge Gained

The imaging-in-endocarditis-flowchart is workable in clinical practice. In patients in whom performance of MDCTA and FDG-PET/CT are suggested in addition to echocardiography by the imaging-in-endocarditis-flowchart, these techniques generally provide relevant complementary diagnostic information, in particular in patients with intracardiac prosthetic material.

## Electronic supplementary material

Below is the link to the electronic supplementary material.

Supplementary material 1 (PDF 413 kb)

Supplementary material 2 (PDF 307 kb)

Supplementary material 3 (PPTX 781 kb)

## Abbreviations

ECG: Electrocardiogram
ESC: European Society of Cardiology
FDG-PET/CT: ^18^F-fluorodeoxyglucose positron emission tomography/computed tomography
ICD: Implantable cardioverter defibrillator
LVAD: Left ventricular assist device
MDCTA: Multi-detector computed tomography angiography
NVE: Native valve endocarditis
PVE: Prosthetic valve endocarditis
TEE: Transoesophageal echocardiography
TTE: Transthoracic echocardiography
