# Supplementary material for: Imaging infective endocarditis: Adherence to a diagnostic flowchart and direct comparison of imaging techniques
Source: J Nucl Cardiol. 2018 Jul 31;27(2):592–608. doi: 10.1007/s12350-018-1383-8 (PMC7174257; doi:10.1007/s12350-018-1383-8)
Supplement: Supplementary file 1 — Supplementary material 1 (PDF 413 kb) [file 12350_2018_1383_MOESM1_ESM.pdf]

Imaging infective endocarditis:

adherence to a diagnostic flowchart and direct comparison of imaging techniques

Journal of Nuclear Cardiology

Anna Gomes, MD[1*]; Peter Paul van Geel, MD/PhD[2]; Michiel Santing, MD[3]; Niek H.J. Prakken, MD/PhD [4]; Mathilde L. Ruis[1,4]; Sander van Assen, MD/PhD[5]; Riemer H.J.A. Slart, MD/PhD[6,7]; Bhanu Sinha, MD/PhD[1]; Andor W.J.M. Glaudemans, MD/PhD[6]

University of Groningen, University Medical Center Groningen, Groningen, Netherlands, Department of: [1]Medical Microbiology, [2]Cardiology, [3]Radiology, [6]Nuclear Medicine&Molecular Imaging.
[4]Carl von Ossietzky University Oldenburg, Oldenburg, Germany. [5]Treant Care Group, Hoogeveen/Emmen/Stadskanaal, Netherlands, Department of Internal Medicine, Infectious Diseases. [7]University of Twente, Enschede, Netherlands, Department of Biomedical Photonic Imaging.

***Correspondence:**

A. Gomes, e-mail:a.gomes@umcg.nl.

Supplementary Table S1: **Final outcome of cardiac diagnosis for endocarditis/ device infection according to TTE/TEE, MDCTA, FDG-PET/CT for each patient included for a head-to-head comparison of imaging modalities (n=46).** AV = aortic valve; Bentall = replacement of aortic valve, aortic root and ascending aorta; bio = biological prosthetic valve implanted; *E. faecalis* = *Enterococcus faecalis*; *L. monocytogenes* = *Listeria monocytogenes;* LVAD = left ventricular device; MDCTA = multidetector computed tomography angiography, scans indicated with an asterisk (*) were indicated by the readers as "possible endocarditis/device infection", scans indicated with the symbol "‡" were suboptimal scans ; mechano = mechanical prosthetic valve implanted; MRSA = Methicillin resistant *Staphylococcus aureus*; MVP = mitral valve plasty; MV = mitral valve; *P. acnes* = *Propionibacterium acnes*; PET = positron emission tomography; PV = pulmonary valve; *S. aureus* = *Staphylococcus aureus*; *S. agalactiae* = *Streptococcus agalactiae; S. dysgalactiae* = *Streptococcus dysgalactiae*; *S. epidermidis* = *Staphylococcus epidermidis*; *S. equi* = *Streptococcus equi; S. gallolyticus* = *Streptococcus gallolyticus*; *S. lugdunensis* = *Staphylococcus lugdunensis*; *S. mitis* = *Streptococcus mitis*; *S. mutans* = *Staphylococcus mutans*; TEE = transesophageal echocardiography; TTE = transthoracic echocardiography; *T. whipplei* = *Tropheryma whipplei;* VSD = ventricular septum defect.

Patient study number	Intracardiac prosthetic material	Pathogen detected	Imaging techniques			Gold standard: expert team diagnosis
			TTE/TEE	MDCTA	PET (visual valve)	
10000077	bio PV	*S. aureus*	Positive	Negative	Positive	Positive
10000079	none	*S. aureus*	Negative	Positive	Negative	Negative
10000080	classic Glenn anostomosis, Blalock-Taussing shunt	none	Negative	Negative	Negative	Negative
10000082	none	*T. whipplei*	Positive	Positive	Positive	Positive
10000087	none	*S. aureus*	Negative	Negative*	Negative	Negative
10000092	pacemaker	*S. aureus*	Negative	Positive	Negative	Positive
10000093	none	*S. aureus*	Negative	Negative‡	Negative	Negative
10000102	bio AV, MVP	*S. aureus*	Positive	Negative	Negative	Negative
10000106	bio AV	*S. gallolyticus*	Negative	Negative	Negative	Negative
10000113	none	*S. aureus*	Negative	Negative	Negative	Negative
10000116	none	*S. aureus*	Negative	Negative	Negative	Positive
10000123	none	*S. aureus*	Negative	Positive*	Negative	Negative
10000125	none	*S. equi*	Negative	Negative*	Negative	Negative
10000126	none	*S. aureus*	Negative	Negative	Negative	Negative
10000129	LVAD, MVP	*S. aureus*	Negative	Positive	Positive	Positive
10000130	none	none	Negative	Negative*	Negative	Negative
10000131	mechano Bentall	*P. acnes*	Positive	Positive	Positive	Positive
10000136	none	*S. aureus*	Negative	Negative	Negative	Negative
10000143	bio AV	*S. mitis*	Negative	Negative	Negative	Negative
10000145	bio AV	*S. aureus*	Positive	Negative	Positive	Positive
10000152	mechano MV	*S. epidermidis*	Positive	Negative‡	Positive	Positive
10000174	none	*S. aureus*	Negative	Negative	Negative	Negative
10000177	none	*S. aureus*	Negative	Positive*	Negative	Negative
10000178	none	*S. aureus*	Negative	Positive	Negative	Negative

10000183	none	none	Negative	Negative	Negative	Negative
10000194	bio Bentall	*L. monocytogenes*	Positive	Positive	Positive	Positive
10000197	none	*S. mitis*	Positive	Positive	Negative	Positive
10000198	none	*T. whipplei*	Negative	Negative*	Positive	Positive
10000204	VSD patch (Goretex)	*S. aureus*	Negative	Negative	Negative	Negative
10000206	none	*S. mitis,* *S. agalactiae,* *S. aureus*	Negative	Negative	Negative	Negative
10000207	none	*S. aureus*	Negative	Negative	Negative	Negative
10000208	none	*S. aureus*	Negative	Positive	Negative	Negative
10000214	none	*S. aureus*	Negative	Negative	Negative	Negative
10000215	none	*S. aureus*	Negative	Negative	Negative	Negative
10000221	MVP	none	Negative	Positive	Positive	Negative
10000242	bio AV	*S. aureus*	Positive	Positive*	Positive	Positive
10000246	bio AV	*E. faecalis*	Positive	Positive	Positive	Positive
10000267	LVAD	*S. lugdunesis*	Negative	Positive	Positive	Positive
10000270	none	*S. aureus*	Positive	Positive	Negative	Positive
10000272	none	*S. aureus*	Negative	Negative	Negative	Negative
10000274	bio AV	*P. acnes*	Positive	Positive	Negative	Positive
10000276	pacemaker	*S. aureus*	Positive	Positive	Positive	Positive
10000282	none	*S. dysgalactiae*	Positive	Negative	Negative	Positive
10000291	mechano MV, pacemaker	*S. epidermidis*	Negative	Negative	Negative	Negative
10000294	none	MRSA	Negative	Negative	Negative	Negative
10000295	none	*S. mutans*	Positive	Positive	Negative	Positive
