# Supplementary material for: Imaging infective endocarditis: Adherence to a diagnostic flowchart and direct comparison of imaging techniques
Source: J Nucl Cardiol. 2018 Jul 31;27(2):592–608. doi: 10.1007/s12350-018-1383-8 (PMC7174257; doi:10.1007/s12350-018-1383-8)
Supplement: Supplementary file 2 — Supplementary material 2 (PDF 307 kb) [file 12350_2018_1383_MOESM2_ESM.pdf]

Imaging infective endocarditis:

adherence to a diagnostic flowchart and direct comparison of imaging techniques

Journal of Nuclear Cardiology

Anna Gomes, MD[1*]; Peter Paul van Geel, MD/PhD[2]; Michiel Santing, MD[3]; Niek H.J. Prakken, MD/PhD [4]; Mathilde L. Ruis[1,4]; Sander van Assen, MD/PhD[5]; Riemer H.J.A. Slart, MD/PhD[6,7]; Bhanu Sinha, MD/PhD[1]; Andor W.J.M. Glaudemans, MD/PhD[6]

University of Groningen, University Medical Center Groningen, Groningen, Netherlands, Department of: [1]Medical Microbiology, [2]Cardiology, [3]Radiology, [6]Nuclear Medicine&Molecular Imaging.
[4]Carl von Ossietzky University Oldenburg, Oldenburg, Germany. [5]Treant Care Group, Hoogeveen/Emmen/Stadskanaal, Netherlands, Department of Internal Medicine, Infectious Diseases. [7]University of Twente, Enschede, Netherlands, Department of Biomedical Photonic Imaging.

***Correspondence:**

A. Gomes, e-mail:a.gomes@umcg.nl.

***Supplementary Table S2:* Cases with any imaging modality positive for the cardiac diagnosis of endocarditis/device infection, as presented in *Supplementary Table S1,* analysed in more depth (n=29).** Grade 1: uptake lower than that of the mediastinum, 2: uptake higher than that of the mediastinum but lower than that of the liver, 3: uptake equal to that of the liver, 4: uptake higher than that of the liver. SUV_{max} is calculated as maximum FDG-uptake at the region of suspected IE divided by the SUV_{mean} FDG-uptake of the thoracic aorta. AML = anterior mitral leaflet, AI = aortic valve insufficiency, AV = aortic valve, IE = infective endocarditis, LA = left atrium, LCC = left coronary cusp, LV = left ventricle, LVOT = left ventricular outflow tract, MI = mitral valve insufficiency, MV = mitral valve, MVP = mitral valve plasty, NCC = non coronary cusp, PFO = patent foramen ovale, PI = pulmonary valve insufficiency, PML = posterior mitral leaflet, PS = pulmonary valve stenosis, PV = pulmonary valve, RCA = right coronary artery, RCC = right coronary cusp, TI = tricuspid valve insufficiency, TV = tricuspid valve.

	Patient study number	TTE/TEE	MDCTA	PET
**1**	10000 077	True positive Several vegetations: 12 mm mobile and spherical on AML which travels into LVOT during systole with MI, PML, and on biological prosthetic PV with PI grade III. Small PFO.	False negative	True positive Grade 2 focal heterogeneous uptake at biological prosthetic PV, with involvement of lymph nodes nearby.
**2**	10000 079	True negative	False positive Mild thickening of AV leaflets. Valvular thickenings AML suspect for vegetation/IE.	True negative
**3**	10000 082	True positive Several vegetations: small, calcified and mobile on all leaflets of calcified AV especially between NCC and RCC with severe AS and AI, non-mobile on calcified MV with moderate MI, 13 mm and mobile on PV with severe PI and slight PS.	True positive AV vegetation and abscess. AV, MV, PV calcification and leaflet thickening.	True positive Grade 4 focal heterogeneous uptake at native AV, with involvement of lymph nodes nearby.
**4**	10000 087	True negative	True negative (false possible): Calcified and thickened AV especially LCC, non-conclusive vegetation/ pannus/ thrombus. Calcification and thickening AML, probably based on pannus, but vegetation/ thrombus not to be ruled out. Possible IE AV/MV.	True negative
**5**	10000 092	False negative	True positive Hypodense foci of thickening and calcification on PML suspect for vegetations/IE. Calcifications and thickening of AV leaflets.	False negative
**6**	10000 102	False positive Dehiscence of MVP, which moves at edges back and forth. Fast decline in between echocardiography's, strong suspicion IE.	True negative	True negative

**7**	10000 116	False negative	False negative	False negative
**8**	10000 123	True negative	False positive (true possible) Focal thickening and calcification AV leaflet not suspect of vegetation but annular thickening highly suspect for IE in the right clinical setting. Thickening and calcification AML non-specific with non-conclusive vegetation/ pannus/ thrombus, possibly IE.	True negative
**9**	10000 125	True negative	True negative (false possible) Thickening of LCC AV including subtle more focal thickening and calcifications with non-conclusive vegetation/ pannus/ thrombus. Possibly degeneration, though IE not to be ruled out.	True negative
**10**	10000 129	False negative	True positive Infection of LVAD outflow graft due to air bubbles with also contrast extravasation and surrounding induration/fluid (possibly leaking out of graft). Retrosternal abscess/hematoma.	True positive LVAD and driveline grade 4 focal heterogeneous visual uptake, with positive nearby lymph nodes.
**11**	10000 130	True negative	False positive (true possible) Heavily calcified AV with excessive thickening of RCC possibly degeneration but with non-conclusive vegetation/ pannus/thrombus, IE not to be ruled out. Minor calcification and thickening MV, no focal thickenings suspect of vegetations. No other signs of IE.	True negative
**12**	10000 131	True positive AV paravalvular cavity that communicates with LVOT, slight paravalvular regurgitation on ventral side of prosthetic AV; no extension towards LA but mobile wall here.	True positive IE prosthetic AV with vegetation, dehiscence, dysfunction, large abscess/ paravalvular leakage. Focal thickening of MV leaflets suspect for vegetations. Dilation of sinotubular junction.	True positive AV grade 4 focal heterogeneous uptake, with mediastinal lymph nodes and 2x abscess formation in the spleen.
**13**	10000 145	True positive Clear signs of MV IE: several vegetations - spherical, calcified, and mobile of 9 mm on AML moving from LV towards LA, and on PML; annulus calcification and moderate MI.	False negative	True positive Grade 4 focal heterogeneous uptake prosthetic AV with lymph node involvement nearby and left groin abscess.
**14**	10000 152	True positive Several small, calcified, and mobile vegetations on prosthetic MV annulus with severe regurgitation.	False negative : suboptimal scan	True positive Prosthetic MV grade 3 focal heterogeneous uptake, with lymph node involvement nearby.
**15**	10000 177	True negative	False positive (true possible) Thickened anterior papillary muscle RV/TV annulus. TV thickening: possible vegetation/ IE. MV leaflet thickening, probably degeneration.	True negative
**16**	10000 178	True negative	False positive Minimal AV calcification and leaflet thickening. MV calcification and focal thickening AML +	True negative

			chordae, suspect of vegetation, DD degeneration. Possible MV IE, no other IE signs.	
**17**	10000 194	True positive Single 8 mm vegetation on LCC of prosthetic AV, with thickened annulus and slight paravalvular regurgitation.	True positive Bio-bentall AV with non-conclusive vegetation/ pannus/ thrombus suspect for IE, with focal, annular thickening and fat infiltration LCC. Mild thickening/calcification MV/PV, with possible vegetation MV. No other signs of IE.	True positive Grade 4 focal heterogeneous uptake at prosthetic AV with involvement of lymph nodes nearby.
**18**	10000 197	True positive Single mobile vegetation of 12 mm at AV, with thickened valve ridges, severe AI, and pericardial fluid.	True positive Minimal calcifications AV, MV. Focal AV leaflet thickening suspect of vegetation/IE, DD degeneration. Possible AV IE, no other signs of IE.	False negative
**19**	10000 198	False negative	False negative (true possible): AV and MV calcification. MV leaflet thickening and minimal non-specific vegetation/ pannus/ thrombus, DD degeneration; possible MV IE. AV leaflet thickening which is degenerative.	True positive MV grade 3 focal heterogeneous uptake.
**20**	10000 208	True negative	False positive Focal thickening AML suspect of vegetation/IE, DD degeneration. No complications of IE. Minor AV calcification and thickening.	True negative
**21**	10000 221	True negative	False positive Possible prosthetic MV IE: focal annular thickening near ring with heavy, partly focal leaflet thickening suspect for IE, DD annular thrombus/ pannus/ degeneration; no complications.	False positive Prosthetic MV grade 4 focal heterogeneous uptake, with involvement of lymph nodes nearby.
**22**	10000 242	True positive Prosthetic AV paravalvular abscess.	True positive (false possible) Prosthetic AV with inconclusive tissue surplus, possibly thrombus/vegetation, with annular infiltration: possible IE.	True positive Prosthetic AV grade 4 focal heterogeneous uptake, with lymph node involvement nearby.
**23**	10000 246	True positive Single 19 mm vegetation PML, atrial side, compact with attached mobile fluttering structure. MV prolapse with systolic anterior movement of AML/chorda in LVOT, and MS. Slight AI, TI, PI.	True positive Calcification and large vegetation MV annulus and leaflets, with MV degeneration and detachment of papillary muscle/chordae: suspect for IE. Suspicion of involvement of the AV annulus due to irregular thickening.	True positive Grade 4 focal heterogeneous uptake at native MV; prosthetic AV equivocal uptake.
**24**	10000 267	False negative	True positive No valvular signs of IE, but possible LVAD infection, infected thrombus/vegetations inside the LVAD cannot be ruled out because of severe metal artefacts.	True positive Grade 4 focal heterogeneous uptake at LVAD, with bone metastatic infection right hip and shoulder, left wrist, and involvement of lymph nodes nearby.
**25**	10000 270	True positive AV single 11 mm vegetation subvalvulary, mobile into LVOT; paravalvular	True positive Possible IE with vegetations AV/ LVOT and AML, and AV leaflet thickening.	False negative

		abscess and fistula. RCC and NCC fusion, NCC extra raphe/sclerosis, slight AS. LCC small and destructed explaining severe AI.		
**26**	10000 274	True positive Multiple vegetations on the lumenal wall of aorta ascendens prosthesis, with ventral of AV a mobile vegetation attached to prosthesis, paravalvular abscess, AI, and pericardial fluid.	True positive AV calcifications, including the aneurysmatic valsalva sinus. Focal thickenings of prosthesis frame, with angulation, RCC dehiscence, and RCC/LCC dilatation. MV calcification and thickening. Findings suspect of IE, DD degeneration.	False negative
**27**	10000 276	True positive Snake-like pacemaker lead vegetation of 20 mm with slight TI. Calcification spot on atrial side of PML, not suspected of vegetation.	True positive Suspicion of thrombus around RV pacemaker lead just below TV, possibly vegetation, though little contrast in RV: to be assessed with ultrasound. No other signs of IE.	True positive Pacemaker lead with grade 2 focal heterogeneous uptake, with metastatic foci in both lungs, bone (spondylodiscitis L2, right hip, right shoulder), aortic root plaque, and RCA.
**28**	10000 282	True positive Single non-mobile 10 mm vegetation above AML or in LVOT. AV calcification with decreased opening, severe AS, and destruction LCC, severe AI, and poor LV function.	False negative	False negative
**29**	10000 295	True positive Several small and mobile fluttery vegetations on AV LCC, largest is 9 mm, with AV perforation. Extensive calcifications on all cusps, thickened, with moderate AI.	True positive AV calcification and non-conclusive vegetation/thrombus with heavy focal thickening, suspect of IE, DD degeneration. No other signs of IE.	False negative
