# Supplementary material for: Imaging infective endocarditis: Adherence to a diagnostic flowchart and direct comparison of imaging techniques
Source: J Nucl Cardiol. 2018 Jul 31;27(2):592–608. doi: 10.1007/s12350-018-1383-8 (PMC7174257; doi:10.1007/s12350-018-1383-8)
Supplement: Supplementary file 3 — Supplementary material 3 (PPTX 781 kb) [file 12350_2018_1383_MOESM3_ESM.pptx]

## Slide 1
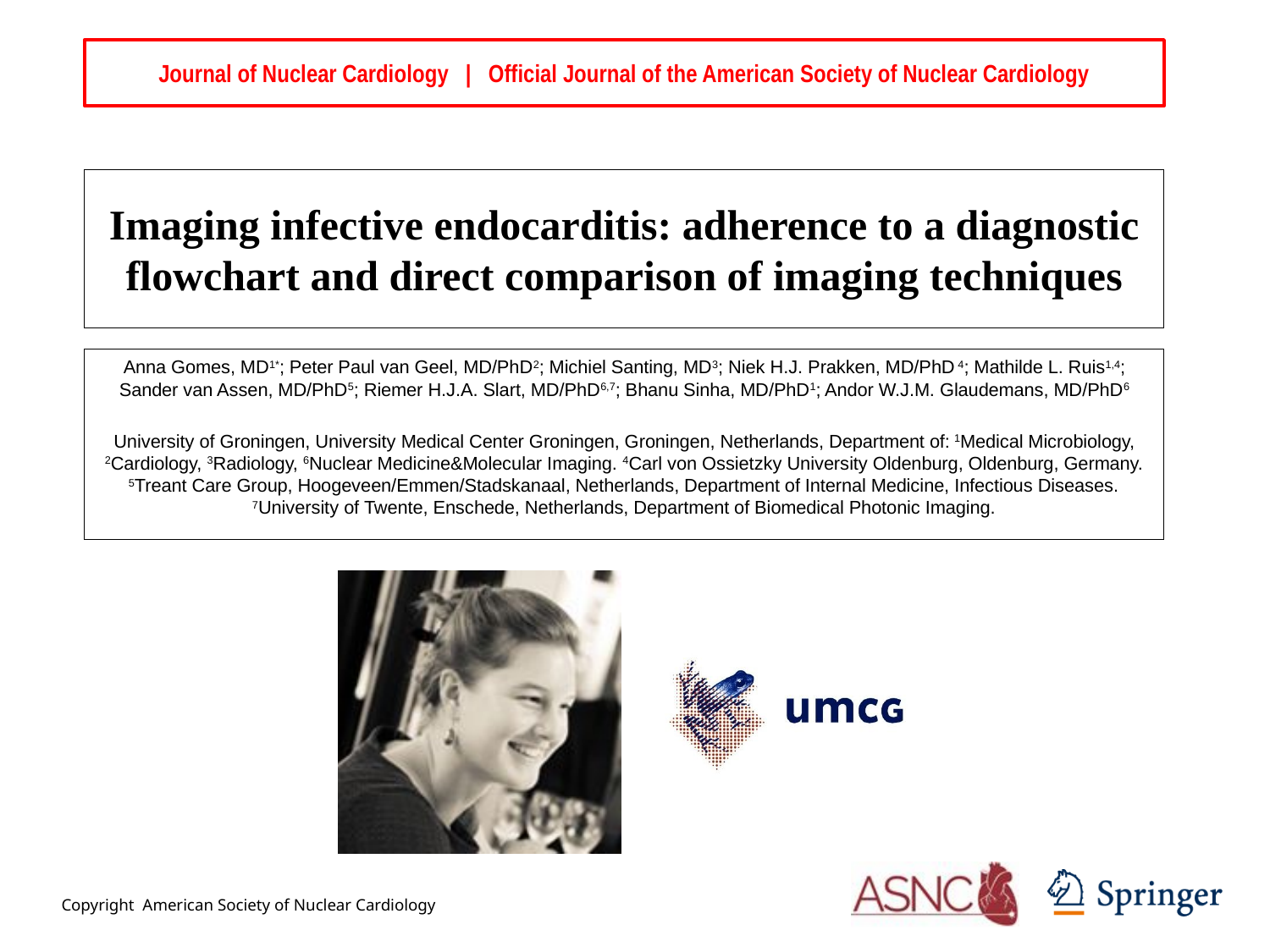

Journal of Nuclear Cardiology | Official Journal of the American Society of Nuclear Cardiology
# Imaging infective endocarditis: adherence to a diagnostic flowchart and direct comparison of imaging techniques
Anna Gomes, MD1*; Peter Paul van Geel, MD/PhD2; Michiel Santing, MD3; Niek H.J. Prakken, MD/PhD 4; Mathilde L. Ruis1,4; Sander van Assen, MD/PhD5; Riemer H.J.A. Slart, MD/PhD6,7; Bhanu Sinha, MD/PhD1; Andor W.J.M. Glaudemans, MD/PhD6
University of Groningen, University Medical Center Groningen, Groningen, Netherlands, Department of: 1Medical Microbiology, 2Cardiology, 3Radiology, 6Nuclear Medicine&Molecular Imaging. 4Carl von Ossietzky University Oldenburg, Oldenburg, Germany. 5Treant Care Group, Hoogeveen/Emmen/Stadskanaal, Netherlands, Department of Internal Medicine, Infectious Diseases. 7University of Twente, Enschede, Netherlands, Department of Biomedical Photonic Imaging.
Copyright American Society of Nuclear Cardiology

## Slide 2
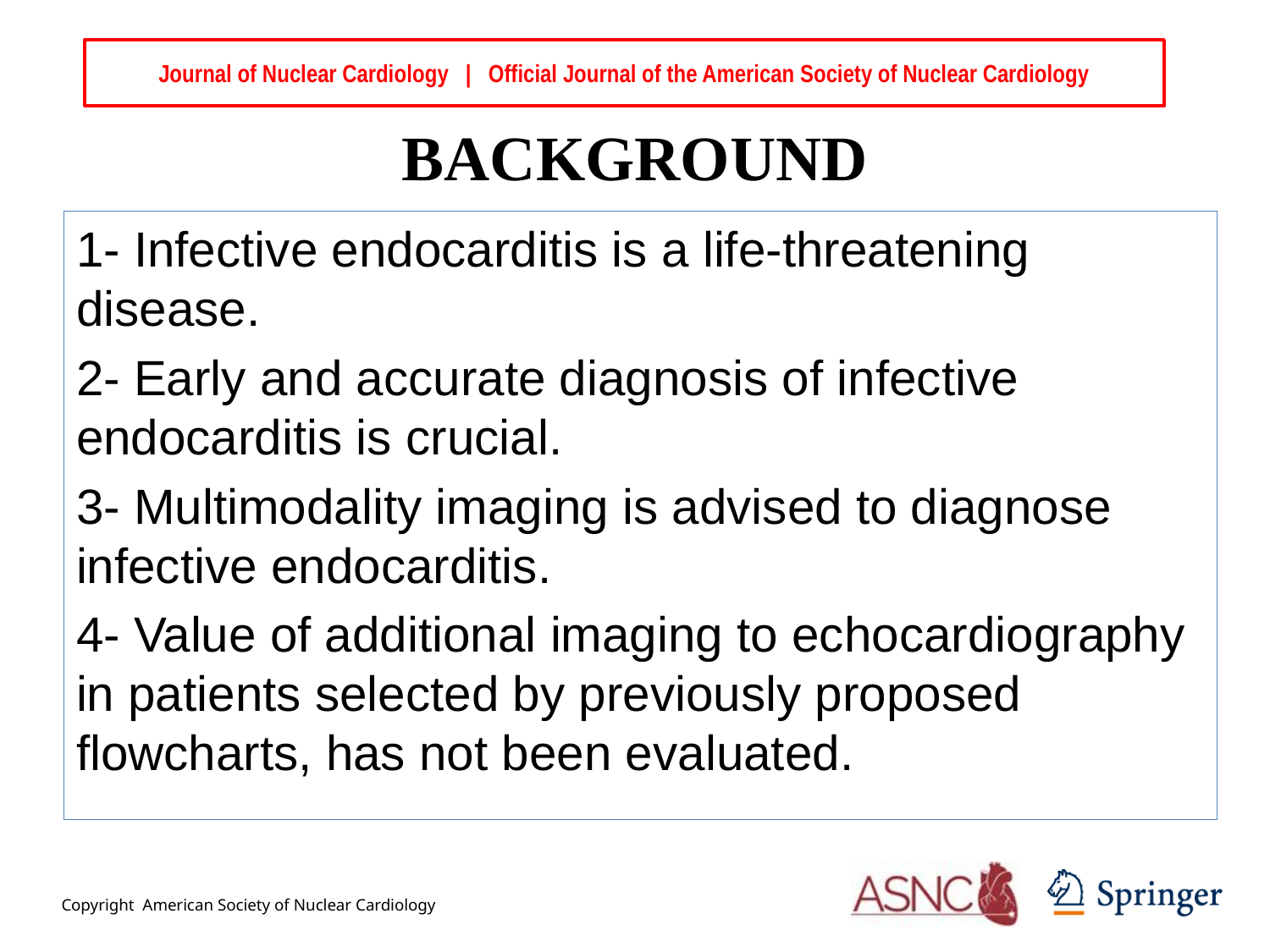

Journal of Nuclear Cardiology | Official Journal of the American Society of Nuclear Cardiology
# BACKGROUND
1- Infective endocarditis is a life-threatening disease.
2- Early and accurate diagnosis of infective endocarditis is crucial.
3- Multimodality imaging is advised to diagnose infective endocarditis.
4- Value of additional imaging to echocardiography in patients selected by previously proposed flowcharts, has not been evaluated.
Copyright American Society of Nuclear Cardiology

## Slide 3
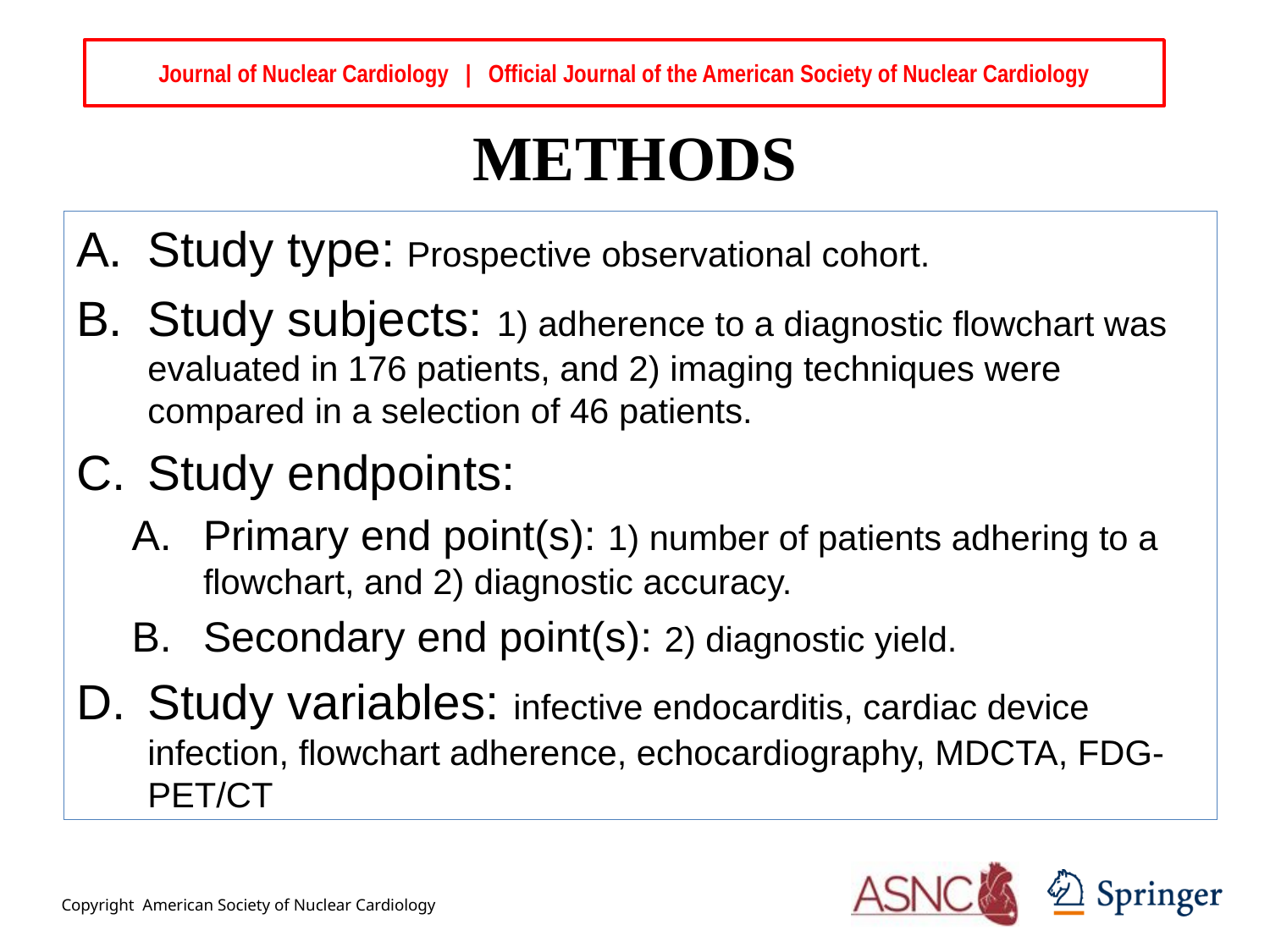

Journal of Nuclear Cardiology | Official Journal of the American Society of Nuclear Cardiology
# METHODS
Study type: Prospective observational cohort.
Study subjects: 1) adherence to a diagnostic flowchart was evaluated in 176 patients, and 2) imaging techniques were compared in a selection of 46 patients.
Study endpoints:
Primary end point(s): 1) number of patients adhering to a flowchart, and 2) diagnostic accuracy.
Secondary end point(s): 2) diagnostic yield.
Study variables: infective endocarditis, cardiac device infection, flowchart adherence, echocardiography, MDCTA, FDG-PET/CT
Copyright American Society of Nuclear Cardiology

## Slide 4
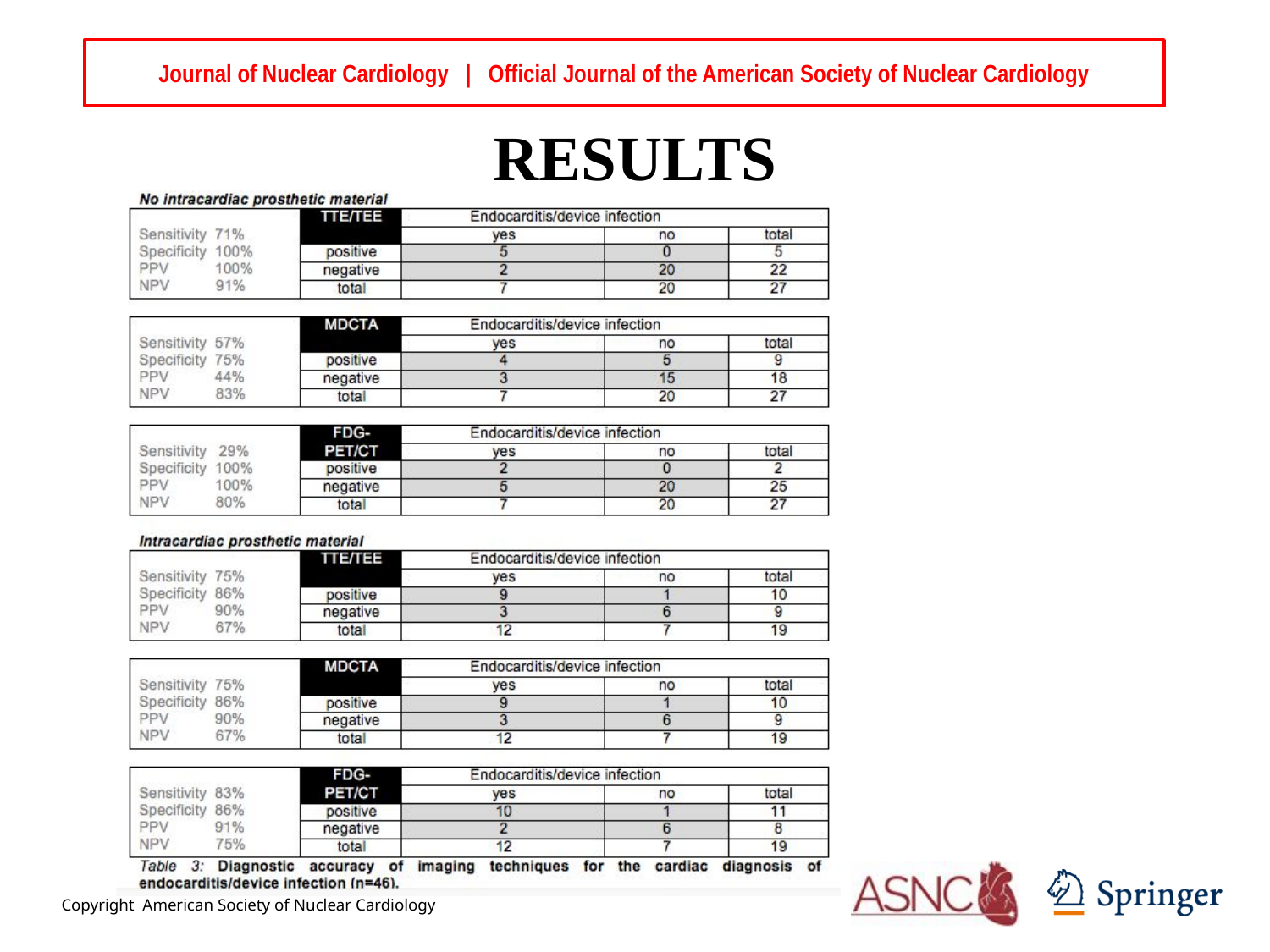

Journal of Nuclear Cardiology | Official Journal of the American Society of Nuclear Cardiology
# RESULTS
Copyright American Society of Nuclear Cardiology

## Slide 5
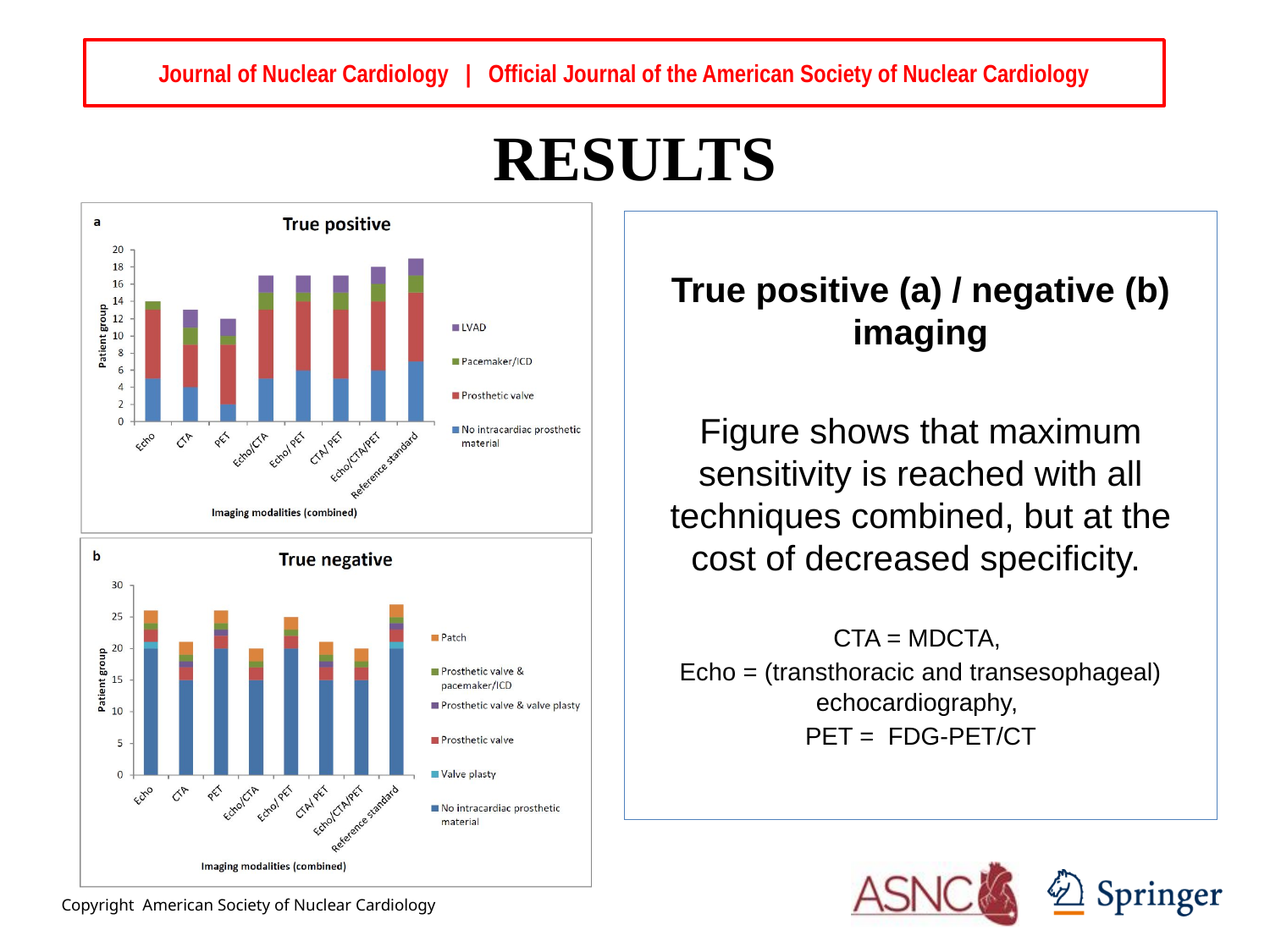

Journal of Nuclear Cardiology | Official Journal of the American Society of Nuclear Cardiology
# RESULTS
True positive (a) / negative (b) imaging
Figure shows that maximum sensitivity is reached with all techniques combined, but at the cost of decreased specificity.
CTA = MDCTA,
Echo = (transthoracic and transesophageal) echocardiography,
PET = FDG-PET/CT
Copyright American Society of Nuclear Cardiology

## Slide 6
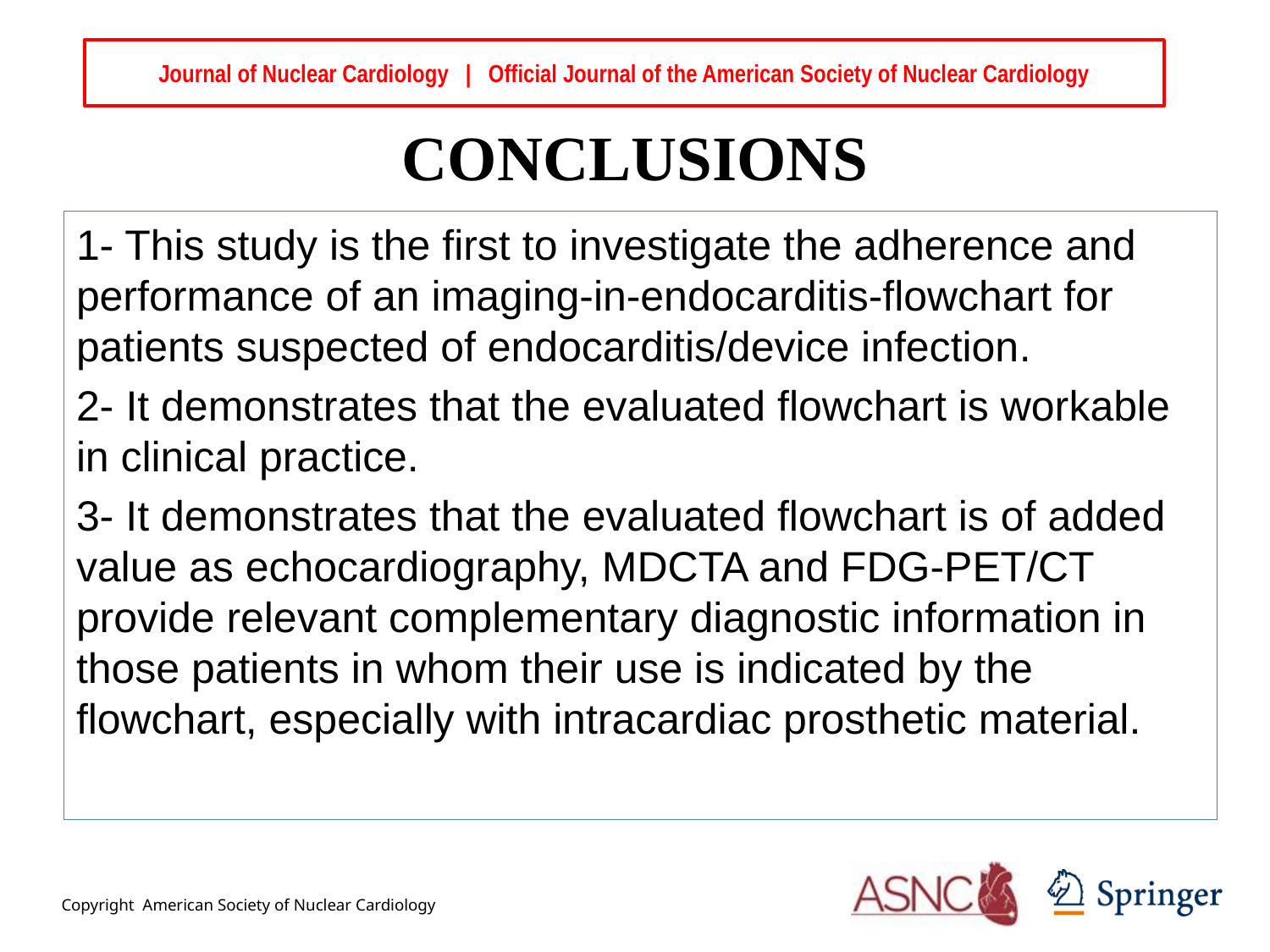

Journal of Nuclear Cardiology | Official Journal of the American Society of Nuclear Cardiology
# CONCLUSIONS
1- This study is the first to investigate the adherence and performance of an imaging-in-endocarditis-flowchart for patients suspected of endocarditis/device infection.
2- It demonstrates that the evaluated flowchart is workable in clinical practice.
3- It demonstrates that the evaluated flowchart is of added value as echocardiography, MDCTA and FDG-PET/CT provide relevant complementary diagnostic information in those patients in whom their use is indicated by the flowchart, especially with intracardiac prosthetic material.
Copyright American Society of Nuclear Cardiology
